# Quantifying resistance to myxomatosis in wild rabbits produces novel evolutionary insights

**DOI:** 10.1017/S0950268823001668

**Published:** 2023-10-12

**Authors:** Brian D. Cooke, Patrick Taggart, Kandarp Patel

**Affiliations:** 1 Institute for Applied Ecology, University of Canberra, Bruce, Australian Capital Territory, Australia; 2 School of Animal and Veterinary Sciences, The University of Adelaide, Roseworthy, South Australia, Australia; 3 Bush Heritage Australia, Melbourne, Victoria, Australia; 4 Department of Primary Industries NSW, Vertebrate Pest Research Unit, Queanbeyan, New South Wales, Australia

**Keywords:** Oryctolagus, rabbit, myxoma virus, virulence, resistance, microevolution, Australia, United Kingdom

## Abstract

Wild rabbits in Australia developed genetic resistance to the myxoma virus, which was introduced as a biological control agent. However, little is known about the rate at which this evolutionary change occurred. We collated data from challenge trials that estimated rabbit resistance to myxomatosis in Australia and expressed resistance on a continuous scale, enabling trends in its development to be assessed over 45 years up to 1995. Resistance initially increased rapidly, followed by a plateau lasting ten years, before a second rapid increase occurred associated with the introduction of European rabbit fleas as myxoma virus vectors. By contrast, in the United Kingdom, where rabbit flea vectors were already present when the myxoma virus initially spread, resistance developed more slowly. No estimates of rabbit resistance to myxomatosis have been made for almost 30 years, despite other highly lethal rabbit pathogens becoming established worldwide. Continued testing of wild-caught rabbits in Australia to determine current levels of resistance to myxomatosis is recommended to assess its current effectiveness for managing pest rabbits. Given the economic and environmental significance of invasive rabbits, it would be remiss to manage such biological resources and ecosystem services poorly.

## Introduction

Wild rabbits *(Oryctolagus cuniculus)* were introduced into Australia by European settlers in the mid-1800s and quickly became major pests, competing with domestic livestock, ruining crops, causing soil erosion, and eliminating native vegetation and wildlife [1]. Nonetheless, in 1950 a highly lethal myxoma virus originating in South American lagomorphs (*Sylvilagus* spp.) was introduced into Australia as a biological control agent. Spread by mosquitoes, it reduced pest rabbit abundance by over 90 per cent and greatly alleviated the problems rabbits caused [1].

However, only two years after the introduction of the virus, Fenner [2] showed that not only had the originally released standard laboratory strain (SLS) given rise to numerous variants, mostly of lesser virulence, but rabbits were also evolving resistance to myxomatosis. Fenner’s definition of resistance, used here, was broad. He defined resistance as ‘the capacity of an animal to resist the ill-effects of an infectious agent’, which generally implies disease tolerance rather than resistance to infection [1]. Fenner defined virulence as ‘the capacity of a virus to cause severe disease or death in a particular host species’ [1].

Using an attenuated field strain virus called KM13 to challenge young adult rabbits collected from the field, it was shown that case fatality rates decreased with successive annual disease outbreaks [3]. Over time, it became necessary to use increasingly virulent viruses including the original SLS virus in challenge trials to demonstrate how the resistance of rabbits had increased.

Rendel [4] became concerned that resistance was increasing so rapidly that soon only the most virulent myxoma viruses available would be able to suppress wild rabbits. He quantified virus virulence and rabbit resistance on a continuous probit scale to emphasise the need for action and asserted that the European rabbit fleas (*Spilopsyllus cuniculi*), then being introduced into Australia as virus vectors [5], should slow the rate at which rabbits developed myxomatosis resistance. His modelling showed, somewhat counterintuitively, that selection for resistance would slow if higher mortality caused by flea-transmitted myxomatosis reduced the proportion of myxomatosis survivors recruited into the breeding population each year [4].

After Fenner’s initial studies on the virulence of the myxoma virus and the resistance of rabbits terminated, other researchers continued to challenge groups of wild rabbits from south-eastern Australia with variants of the myxoma virus to assess their increasing resistance (e.g., [1, 6–10]). These challenge trials provided continuous information on the evolution of myxomatosis resistance in wild rabbits spanning 45 years (1950–1995). During the 1990s, such work was largely discontinued because of animal welfare considerations and less interest in myxomatosis research after rabbit haemorrhagic disease virus (RHDV) was introduced into Australia as an additional biocontrol agent. Nonetheless, work of this kind is still possible if animal welfare concerns are addressed [10].

Here, we have assembled the results from those earlier challenge trials and used Rendel’s [4] method to quantify changes in myxomatosis resistance in rabbits over the 1950–1995 period. We show that although rabbit resistance increased over the long term, the rate of this increase varied considerably. Rabbit resistance to myxomatosis increased most rapidly when the virus was first introduced; this rate of increase then slowed before increasing once again. We discuss the reasons behind this variable rate of evolution and build a case that the second increase in resistance was due to the introduction of European rabbit fleas, which heightened the impact of myxomatosis by spreading it among highly susceptible young rabbits in winter when low temperatures increased mortality [11]. We also consider the advantages of using Rendel’s method to estimate rabbit resistance to myxomatosis on a continuous scale and its potential for facilitating comparisons of the evolutionary history of myxomatosis resistance in both Australia and Europe. Finally, we consider the importance of our results for managing pest rabbits in Australia and recommend continuing studies of the co-evolution of virus and host to support future rabbit management.

## Methods

### Rendel’s method for quantifying myxomatosis resistance

Rendel [4] published his method for quantifying myxomatosis resistance in a journal that no longer exists, and copies of the article are not easily obtained. Hence, we provide a detailed explanation.

Rendel assumed that a theoretical myxoma virus killed 50% of unselected wild rabbits and made this the origin or baseline for a continuous probit resistance scale. Using case fatality rates observed in wild rabbits during experimental myxoma challenge trials, he then placed other trial results in a relative position on the scale according to the number of standard deviations (σ) the calculated probit value was from the theoretical baseline virus, assuming a normal distribution around observed case fatality rates. For example, as the attenuated KM13 virus killed 88% of unselected rabbits, Rendel placed KM13 at 1.2 σ (i.e. 6.18 (probit value at 88%) – 5.00 (probit value at 50%) = 1.18 rounded to 1.2 σ) above the baseline. From challenge trials comparing KM13 and SLS virus, SLS was subsequently given a value of 3.4 σ above the origin.

Rendel then showed how the case fatality rates of rabbits could also be converted to probits on a continuous scale, adjusting each according to the virulence of the challenge virus used. For example, in 1961 SLS virus killed 71% of rabbits from Ouyen in the Victorian Mallee [6] enabling the resistance of those rabbits to be calculated at 0.55 (5.55 (probit value of 71%) – 5.0 (probit value of 50%) = 0.55 σ) of a standard deviation below the assigned virulence of SLS, that is 3.4 – 0.55 = 2.85 σ above unselected rabbits.

Over time, where groups of partially resistant rabbits from the same locality were challenged with different virus variants, virulence estimates were assigned to Glenfield myxoma virus = 4.7 σ and Lausanne (Lu) myxoma virus = 5.2 σ. This allowed us to place data from additional challenge trials on wild rabbits on the scale so that changes in resistance could be followed beyond the limited time and over a larger scale of virulence and resistance change than Rendel had been able to consider.

### Collation of data from challenge trials

Beginning with Fenner’s initial experiments [3], we searched the literature for challenge trials of wild rabbits in Australia in which KM13, SLS, Glenfield, or Lausanne viruses had been used [1, 6–10].

There were few reports of equivalent trials from Europe except for a series of trials in the United Kingdom (UK) [12–15]. Based on Ross and Sanders [13], the Brecon virus used in most challenge trials was assigned a virulence of 2.33 σ because it killed all but one of 96 laboratory rabbits (99% mortality). The virulence values of the Cornwall virus and Wiltshire virus used in other challenge trials for which data were available were derived from references [3] and [15], respectively.

Not all references listed the number of rabbits used in each challenge trial. This prevented the weighting of observations according to sample size in subsequent analyses. However, in instances where the test viruses killed all rabbits, the number of rabbits in each trial was provided. Following Miller and Tainter [16], we used this sample size to estimate the maximum likely value for resistance. For example, where Lausanne killed all 10 rabbits (case fatality of 100%) in a challenge trial the corrected case fatality % using 10 as n in the formula (100*(1 − (0.25/n)) is 98%. This corresponds to a rabbit resistance of 7.05–5.00 = 2.05 σ below the probit virulence of the challenge virus.

### Regions where rabbits for challenge trials were obtained

#### Australia

Rabbits used in challenge trials came from south-eastern Australia and predominantly from within the Murray–Darling River Basin. Nonetheless, several large samples also came from Gippsland in eastern Victoria. Only one group of rabbits came from the arid Lake Eyre Basin.

The climate varies across this part of Australia from sub-alpine to arid, although most sites where rabbits were collected had a Mediterranean-like climate with warm dry summers and cool wet winters. For analyses, average annual rainfall was used as the simplest variable that could explain variations in rabbit resistance associated with the gradient from the cool wet to warm dry habitats. Monthly minimum temperatures were also assessed as possible explanatory variables influencing the intensity of winter myxomatosis outbreaks among largely nocturnal rabbits.

#### United Kingdom

Rabbits that were challenged in the UK were mostly from Norfolk, with additional samples from the counties of Hampshire, Wiltshire, Angus (Scotland), and the island of Skokholm (Wales). All sites have a similar humid temperate oceanic climate. Supplementary Material, Table S1 contains all UK and Australian challenge trial data.

#### Data analysis

All statistical analyses were performed in R (v 4.1.1) statistical software [17].

Using case fatality rates from each of the compiled challenge trials, we calculated corresponding probit values. These values represented the rabbits’ resistance to myxomatosis; see the above description. Using generalised linear regression, we then modelled the resistance of Australian rabbits to myxomatosis (outcome) as a function of years since SLS release, average annual rainfall (mm) and average minimum temperature (°C) for the coldest month at the site of origin of the rabbits, and the myxoma virus strain used in each challenge trial. We included a b-spline for years since SLS release using the *bs (‥, df = 4)* function from the splines package [18] as the development of rabbit resistance to myxomatosis through time was expected to be non-linear, consistent with that observed in descriptive plots. We additionally trialled the inclusion of log (years since SLS release) as Zhang and Hill [19] and Kerr et al. [20] have argued that the response to selection for a certain trait slows with progressive generations due to costs to selection, as in the case of rabbits that were selected for increased production of young showing less resilience to disease [21]. All other predictor variables were included in the models as standard, parametric fixed effects. Similarly, we modelled the relationship between rabbit resistance to myxomatosis in the UK and years since myxoma virus arrival using a generalised linear regression model. We compared model parsimony using the Akaike information criterion corrected for small sample sizes (AICc) [22] and visually assessed model fit using the plot function in the base package of R.

### Independent validation of model outcomes

It was unavoidable that the collection of Australian data involved using viruses of increasing virulence in challenge trials as rabbit resistance increased. Consequently, the effects of years since release and virus virulence could potentially be confounded if any of the challenge viruses used had phenotypic differences affecting mortality that departed greatly from each other. To check that our results were realistic, we compared changes in genetic resistance with published trends in indices of rabbit abundance, based on spotlight counts in Australia and national hunting bag surveys in the UK, anticipating that rabbit populations should only begin to recover as disease resistance increased substantially.

## Results

We collated data from 37 separate challenge trials in Australia. Data from one challenge trial involving rabbits from a sub-alpine region were excluded from the analysis because they had a disproportionate influence on model fit.

Our most parsimonious model for Australian data included a spline for rabbit resistance to myxomatosis and a parametric, fixed effect term for annual average rainfall for the origin of the challenged rabbits (Table 1; Supplementary Material, Table S2). We found limited evidence for the effects of virus strain or variant or average minimum monthly temperature on rabbit resistance to myxomatosis.

**Table 1.** Model selection summary table for rabbit resistance to myxomatosis in Australian rabbits

Model covariates	No. of parameters	Log likelihood	AICc	∆AICc	AICc wt	Cumulative AICc wt
bs (years since SLS release), average annual rainfall	7	−33.32	83.18	–	0.9051	0.9051
bs (years since SLS release), average annual rainfall, myxoma virus strain	10	−31.35	88.07	4.89	0.0784	0.9836
bs (years since SLS release), average annual rainfall, myxoma virus strain, average minimum temperature of coldest month	11	−31.34	91.27	8.09	0.016	0.9994
bs (years since SLS release), average annual rainfall, average minimum temperature of coldest month	5	−43.84	98.99	15.82	0.0003	0.9997
log (years since SLS release), average annual rainfall	4	−45.59	100.03		0.0002	0.9999
log (years since SLS release), average annual rainfall, average minimum temperature of coldest month	5	−45.59	102.48		0.0001	0.9999
log (years since SLS release), average annual rainfall, myxoma virus strain	7	−43.35	103.24		0.0000	1.0000
log (years since SLS release), average annual rainfall, myxoma virus strain, average minimum temperature of coldest month	8	−43.09	105.5		0.0000	1.0000

**Figure 1.**
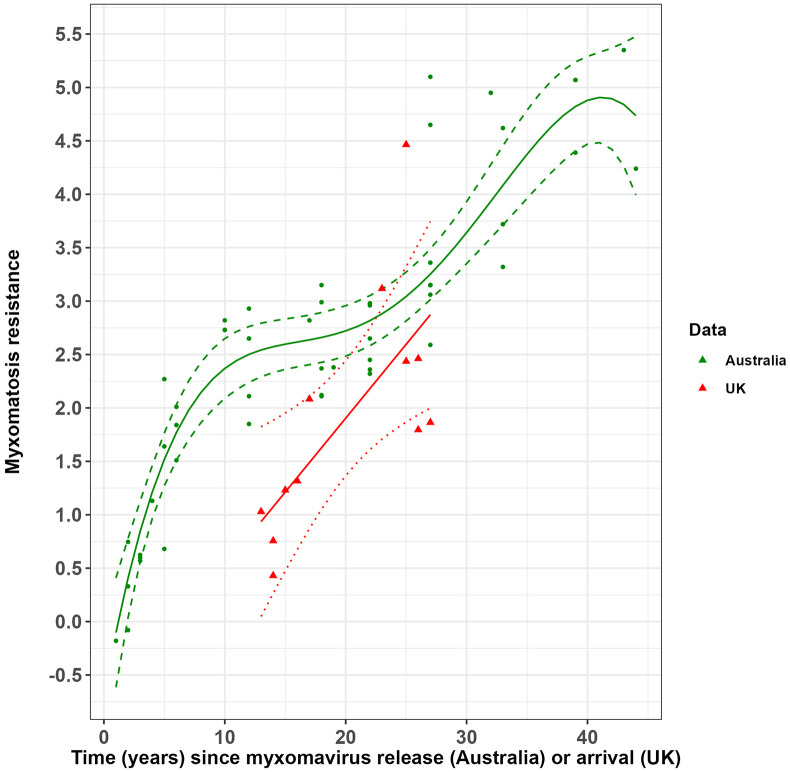
Change in rabbit resistance to myxomatosis since the release of the standard laboratory strain of myxoma virus (SLS) in Australia and since the arrival of myxoma virus in the UK. The solid green line and dashed green lines represent the predicted relationship between resistance in Australian rabbits to myxomatosis and years since myxoma virus (SLS strain) release and associated 95% confidence intervals. Solid red and dotted red lines represent the predicted relationship between resistance in UK rabbits to myxomatosis and years since myxoma virus arrival with associated 95% confidence intervals. Green points and red triangles represent the raw challenge trial data from Australia and the UK, respectively.

The accumulation of resistance to myxomatosis in Australian rabbits through time, following the initial release of SLS, was non-linear (Figure 1). Rabbit resistance rose rapidly in the initial 10 years after the release of SLS, before reaching a plateau lasting for a further 10–15 years. This plateau was then followed by a sharp rise in rabbit resistance to myxomatosis after the establishment of European rabbit fleas in 1975, 25 years after SLS release.

**Figure 2.**
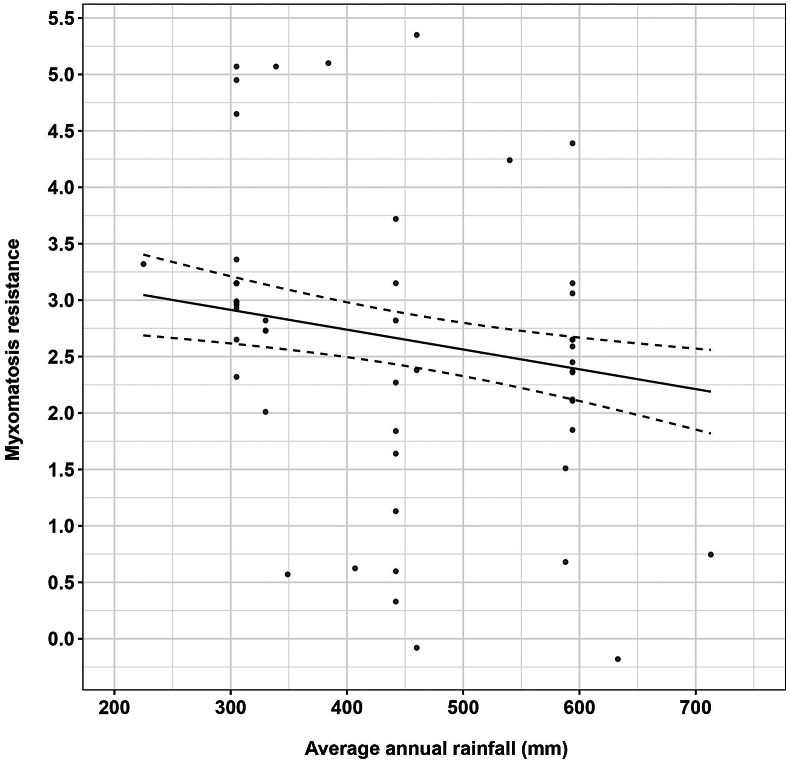
Changes in myxomatosis resistance in Australian rabbits over rainfall gradient. The solid black line and dashed black lines represent the relationship between myxomatosis resistance in Australian rabbits and average annual rainfall and associated 95% confidence intervals. Points represent the raw challenge trial data.

Annual average rainfall had a significant negative linear relationship with the development of rabbit resistance to myxomatosis in Australia (Figure 2). Rabbit resistance to myxomatosis was highest at sites with 200–300 mm average annual rainfall and slowly decreased with increasing rainfall.

Rabbit resistance to myxomatosis in the UK increased significantly with time (years) since the virus’ arrival (Figure 1 and Supplementary Material, Table S3). Resistance in UK rabbits was approaching that of Australian rabbits about 25 years after the myxoma virus arrived.

### Validation of model outcomes

**Figure 3.**
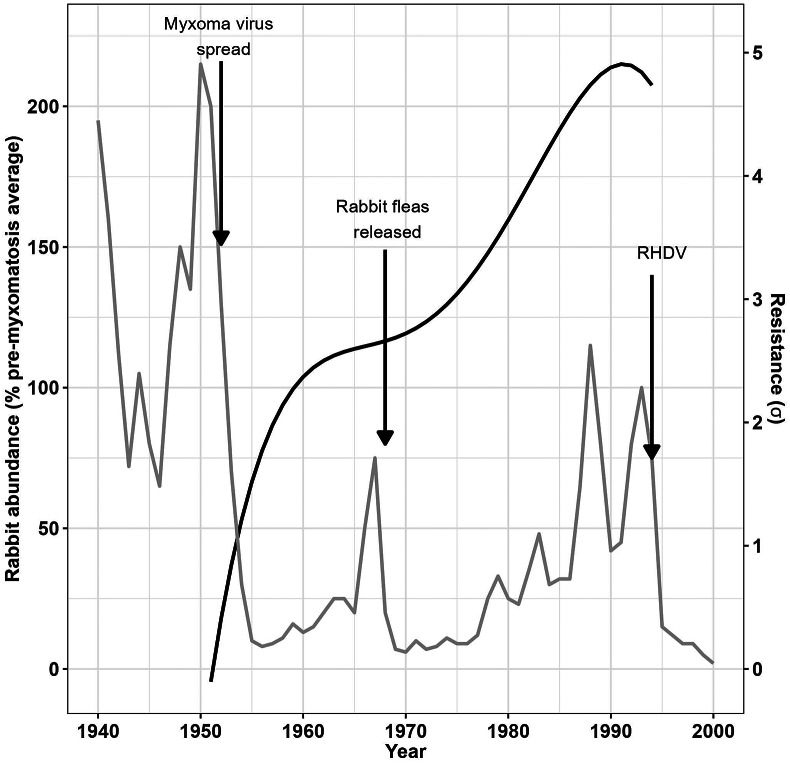
Rabbit population size in relation to rabbit resistance to myxomatosis. The build-up of resistance to myxomatosis in wild rabbits in Australia (solid black line) explains the partial recovery of the rabbit population (grey line) following the initial introduction of the myxoma virus and the subsequent introduction of European rabbit fleas before the introduction of rabbit haemorrhagic disease virus (RHDV) again reduced rabbit abundance.

The two phases during which myxomatosis resistance in Australian wild rabbits increased corresponded with distinct changes in rabbit population size as recorded in previously published data on rabbit population dynamics [23] (Figure 3). The rabbit population fell dramatically following the initial release of the myxoma virus, increased as rabbits became more resistant to myxomatosis, and fell again following the introduction of rabbit fleas before increasing once again.

Published data on long-term trends in rabbit abundance are also available for the UK [24], and these support our limited observations (Figure 1) that rabbits developed resistance to myxomatosis more slowly than in Australia. After an initial population crash, no sharp increase in rabbit abundance in the UK was seen until about 1990 when rabbits apparently gained sufficient resistance to overcome flea-borne myxomatosis (data not shown).

## Discussion

The experimental data from Australian rabbits show that resistance to myxomatosis increased rapidly during the first 10 years (1950–1960) following the release of the SLS virus. The rate of evolution of resistance then slowed, and there was little further gain until a decade later (1970s) when the rate of evolution of resistance increased once again.

The initial rapid increase in resistance in Australian rabbits in the face of mortality of over 99% caused by the myxoma virus is well understood [2]. However, the intermediate period of low selection for resistance cannot be readily explained by interactions between the myxoma virus and the rabbit host alone. Williams and Parer [25] estimated that during annual myxomatosis outbreaks at Lake Urana from 1968 to 1971, 75% of young rabbits older than 3 months became infected with the myxoma virus and 60% recovered. According to Table 2 in Rendel [4], that should have resulted in continuing moderate selection for resistance. However, our model suggests that rabbit resistance increased very slowly at that time.

When the SLS myxoma virus was first released in Australia and it killed an extremely high percentage of rabbits, any rabbit that survived the disease would have subsequently benefited from a density-dependent effect on reproduction. Reduced rabbit numbers following disease outbreaks would mean ample food resources with little competition for the surviving animals, leading to increased reproductive output and maximum recruitment of resistant progeny [26]. Nonetheless, as rabbit abundance increased again, recruitment of young rabbits that survived myxomatosis would be reduced because they had to contend with increasingly overgrazed, poor-quality pasture. Indeed, Williams and Parer [25] recorded that in the late 1960s 20% of the sub-adult rabbits at Lake Urana contracted myxomatosis and recovered but died soon after from an apparent food shortage.

The alternative explanation for the change in myxomatosis resistance through time in Australian rabbits was that genetic rearrangement became more complex or costly [19, 20, 21], and hence, the development of resistance slowed through time. However, we found little support for this idea, which may explain the initial rapid increase and slowing of the development of resistance but cannot explain the subsequent increase in resistance observed.

In the UK, the build-up of resistance to myxomatosis followed a quite different trajectory than that seen in Australia (Figure 1). Nonetheless, the resistance of UK rabbits was approaching that of Australian rabbits about 25 years after the myxoma virus arrived.

### Resistance increased in association with the introduction of European rabbit fleas into Australia

After the decade when rabbit resistance to myxomatosis increased slowly, resistance began to increase rapidly again in the late 1960s, and populations of rabbits with high resistance became common after 1975. This corresponds with the first releases of European rabbit fleas from 1968 onwards and their widespread distribution by 1975 [11].

European rabbit fleas became well-established throughout Gippsland and most of the Murray–Darling River Basin, and our data mostly came from within that area. An observation from Quinyambie in the Lake Eyre Basin was the only exception because the site is far too arid for European rabbit fleas to persist [11, 27]. Indeed, in the late 1980s, rabbits from Quinyambie still had low resistance to the Lausanne virus compared with rabbits from other sites where rabbit fleas were present [7].

The outlying observation from Snowy Plains rabbits that was excluded from our data set because of extremely high average annual rainfall is likewise of interest. Despite the general trend for rabbits in high rainfall areas to be less resistant than those in drier areas (this study), the high resistance of Snowy Plains rabbits (4.62 σ) might be explained if the establishment of rabbit fleas had led to such a sharp increase in rabbit resistance that the former negative correlation between average annual rainfall and resistance to myxomatosis was changing at the time challenge tests were done in 1984.

In the UK, myxoma variants derived from the highly virulent Lu virus were spread from France in 1953 [13]. Because European rabbit fleas were already present and mosquitoes were relatively unimportant as vectors [28], this was unlike the situation in Australia where SLS, a myxoma virus of lesser virulence, carried by mosquitoes, was introduced first and rabbit fleas became widely established only 18–25 years later.

As Rendel [4] thought, selection for resistance to myxomatosis may occur more slowly where fleas are the main vector and rabbits experienced increased mortality. This is supported by field observations that mortality is very high when European rabbit fleas transmit the myxoma virus among highly susceptible young rabbits in winter [11]. Consequently, rabbit populations in the UK might have persisted initially because some rabbits were able to avoid contracting the disease [29, 30]. With few rabbits that survived myxomatosis being recruited into the rabbits’ breeding population relative to animals that avoided infection, the accumulation of resistance genes would be reduced [4]. The rate of increase in resistance in Australian rabbits after European rabbit fleas were introduced was slower than the initial rate of increase when mosquitoes were the main vector, and it approximated that of UK rabbits (Figure 1).

### Implications for understanding changes in myxoma virus virulence and genetics

It is now widely considered that myxoma virus virulence and rabbit resistance are co-evolving [10, 31]. However, this observed process of co-evolution does not accord with the Red Queen hypothesis of Van Valen [32] or a strict biological arms race of Dawkins and Krebs [33] where increased resistance in rabbits leads to increased virulence of the virus and, in turn, this leads to selection for greater resistance. Instead of increasing steadily, as might be expected if increases in resistance were driven predominantly by a virus–host interaction, rabbit resistance in Australia increased in a stepwise fashion. This demonstrates the critical importance of factors external to the rabbits and the virus themselves, in this case the interplay between vectors, population density, and recruitment determined by the food supply, in driving the evolution of virus–host relationships [11].

Furthermore, after 1974, myxoma viruses in Australia split into two recognisable lineages, a and b [10]. This might also be a consequence of the establishment of European rabbit fleas and may be the result of the disruptive selection of myxoma viruses because the fleas became established only in part of the rabbits’ distribution, where rainfall is >200–250 mm annually [11]. Viruses might then have been selected for their ability to persist in areas with and without European rabbit fleas. In short, there are several lines of evidence suggesting that the release of European rabbit fleas not only influenced the evolution of rabbit resistance and myxoma virus virulence but also caused a change in the virus independently of a presumed biological arms race.

### Advantages of using Rendel’s method for assessing virulence and resistance

Expressing virus virulence and rabbit resistance on continuous probit scales as Rendel [4] suggested enabled us to analyse trends in the evolution of resistance in rabbits in a far more detailed way than could be achieved by inspecting data on the case fatality rates of groups of rabbits challenged with different myxoma viruses. Additionally, by expressing rabbit resistance data from both Australia and the UK on the same probit scale, differences in the trajectories of the evolution of myxomatosis resistance in each country can be demonstrated (Figure 1).

Until now, investigations of rabbit resistance to myxomatosis have mostly been viewed from medical or veterinary perspectives, but Rendel’s method also aligns this research with a broader range of microevolutionary studies. Because rabbit resistance is expressed in standard deviations, it can be readily converted to haldanes (a change in one standard deviation per generation of a physical or physiological characteristic such as wing length or immune response [34]) widely used to compare rates of microevolution across groups of different plant and animal species [35].

For example, Australian data show that rabbit resistance departed from the origin by 2.37 σ (CI 2.08–2.64) within the first 10 years after the release of the myxoma virus. Because the generation time of rabbits is approximately one year (i.e. average age of reproductive rabbits is about a year [36]), this represents an average rate of evolution of between 0.21 and 0.26 haldanes, comparable to microevolution rates seen in other natural animal populations subject to intense selection [35]. Data from the UK are limited but suggest a slower rate of evolution of 0.14 haldanes as Rendel [4] had anticipated.

### Relevance of findings to other fields

Our results demonstrate that Rendel’s [4] approach for quantifying changes in host resistance to pathogens can be applied in related fields, especially evolutionary biology. Rabbit resistance evolved in steps, indicating that it is a good example of punctuated evolution [37] and the virulence of the co-evolving myxoma virus shows similar trends [38]. This makes the myxoma virus–rabbit model a useful starting point for exploring comparable data on zoonotic diseases such as vesicular stomatitis and severe acute respiratory syndrome (SARS).

## Conclusion and recommendations

We have shown how scattered and often limited experimental information on the evolution of rabbit resistance to myxomatosis can be brought together in a meaningful way. The results point to the introduction of European rabbit fleas as a major factor influencing the rate of increase in myxomatosis resistance in Australia. This is an alternative to the Red Queen hypothesis [32] where increases in rabbit resistance are expected to be driven by a biological arms race in which virus virulence increases to match rabbit resistance and, in turn, rabbit resistance again increases.

The initial rapid increase in rabbit resistance in Australia followed by a period of relative stability before increasing rapidly again is difficult to reconcile simply in terms of rabbit–virus interactions. Instead, it points to the need to keep in mind the idea that ecological processes and interactions also play a key role in epidemiology and evolution.

It is now almost 30 years since any estimates of rabbit resistance to myxomatosis were made. In that time, two forms of rabbit haemorrhagic disease virus (RHDV and RHDV2) have spread among wild rabbits in Australia, yet it is not known whether that led to a further increase or a decrease in myxomatosis resistance. If myxomatosis now occurs infrequently, or increased resistance to RHDV and RHDV2 has come at a cost to myxomatosis resistance, rabbits might now have reduced resistance to myxomatosis, which would be some assurance that rabbit resistance is not likely to draw far ahead of virus virulence and severely reduce the efficacy of biological control.

There is a need for ongoing studies on myxomatosis including experiments to assess rabbit resistance and current virus virulence. The biological control of rabbits is worth well over A$ 1 billion annually to Australia’s rural industry economy [39]; hence, the need for such research cannot be put aside. It would be remiss to manage these biological resources and ecosystem services poorly.

## Supporting information

Cooke et al. supplementary material Cooke et al. supplementary material

## Data Availability

The raw data used in this study are contained within the Supplementary Data Files available on the Cambridge Core website.

## Supplementary material

For supplementary material accompanying this paper visit http://doi.org/10.1017/S0950268823001668. click here to view supplementary material
