# Supplementary material for: Quantifying resistance to myxomatosis in wild rabbits produces novel evolutionary insights
Source: Epidemiol Infect. 2023 Oct 12;151:e182. doi: 10.1017/S0950268823001668 (PMC10644057; doi:10.1017/S0950268823001668)
Supplement: Cooke et al. supplementary material [file S0950268823001668sup001.docx]

'*Epidemiology and Infection*'

Quantifying resistance to myxomatosis in wild rabbits produces novel evolutionary insights.

B.D. Cooke, P. Taggart and K.K. Patel

'Supplementary Material'

Tables S1 – S3.

Table S1. Raw data collated from the trials in Australia and the UK.

| Country | Year | Years since myxoma virus release/arrival | Rabbit source location | Average annual rainfall(mm) | Average minimum temperature of coldest month | Strain | Case fatality rate(%) | Resistance to myxomatosis (probit scale) | Reference  [Corresponding reference in main document] |
| --- | --- | --- | --- | --- | --- | --- | --- | --- | --- |
| Australia | 1952 | 2 | Various sources | 460 | 3.1 | KM13 | 90 | -0.08 | Fenner and Fantini 1991 [1] |
| Australia | 1953 | 1 | Yarram | 633 | 4.5 | KM13 | 96 | -0.18 | Marshall and Fenner 1958 [3] |
| Australia | 1953 | 2 | Albury | 713 | 2.7 | KM13 | 74 | 0.75 | Marshall and Fenner 1958 [3] |
| Australia | 1953 | 3 | Noorong | 349 | 4.1 | KM13 | 81 | 0.57 | Marshall and Fenner 1958[3] |
| Australia | 1953 | 3 | Barrenbox | 407 | 2.9 | KM13 | 79 | 0.62 | Marshall and Fenner 1958 [3] |
| Australia | 1953 | 2 | Urana | 442 | 3.2 | KM13 | 88 | 0.33 | Marshall and Fenner 1958 [3] |
| Australia | 1954 | 3 | Urana | 442 | 3.2 | KM13 | 80 | 0.6 | Marshall and Fenner 1958 [3] |
| Australia | 1955 | 4 | Urana | 442 | 3.2 | KM13 | 54 | 1.13 | Marshall and Fenner 1958[3] |
| Australia | 1956 | 5 | Urana | 442 | 3.2 | KM13 | 33 | 1.64 | Marshall and Fenner 1958 [3] |
| Australia | 1956 | 5 | Urana | 442 | 3.2 | SLS | 87 | 2.27 | Marshall and Fenner 1958 [3] |
| Australia | 1956 | 5 | Maryvale | 588 | 2.6 | KM13 | 70 | 0.68 | Marshall and Fenner 1958 [3] |
| Australia | 1957 | 6 | Urana | 442 | 3.2 | KM13 | 26 | 1.84 | Marshall and Fenner 1958 [3] |
| Australia | 1957 | 6 | Maryvale | 588 | 2.6 | KM13 | 38 | 1.51 | Marshall and Fenner 1958 [3] |
| Australia | 1957 | 6 | Ouyen | 330 | 4.3 | KM13 | 21 | 2.01 | Marshall and Fenner 1958 [3] |
| Australia | 1961 | 10 | Ouyen | 330 | 4.3 | SLS | 75 | 2.73 | Douglas 1962 [6] |
| Australia | 1961 | 10 | Ouyen | 330 | 4.3 | SLS | 71 | 2.73 | Douglas 1962 [6] |
| Australia | 1961 | 10 | Ouyen | 330 | 4.3 | Glenfield | 97 | 2.82 | Douglas 1962 [6] |
| Australia | 1964 | 12 | Mallee | 305 | 3.1 | SLS | 68 | 2.93 | Shepherd and Edmonds [1] |
| Australia | 1964 | 12 | Mallee | 305 | 3.1 | Glenfield | 98 | 2.65 | Shepherd and Edmonds [1] |
| Australia | 1968 | 17 | Urana | 442 | 3.2 | SLS | 72 | 2.82 | Sobey et al. 1970 [7] |
| Australia | 1969 | 18 | Mallee | 305 | 3.1 | SLS | 66 | 2.99 | Shepherd and Edmonds [1] |
| Australia | 1969 | 18 | Mallee | 305 | 3.1 | Glenfield | 94 | 3.15 | Shepherd and Edmonds [1] |
| Australia | 1970 | 19 | Not known | 460 | 3.1 | KM13 | 12 | 2.38 | Sobey et al. 1970 [7] |
| Australia | 1974 | 22 | Mallee | 305 | 3.1 | SLS | 67 | 2.96 | Shepherd and Edmonds [1] |
| Australia | 1974 | 22 | Mallee | 305 | 3.1 | Glenfield | 96 | 2.98 | Shepherd and Edmonds [1] |
| Australia | 1974 | 22 | Mallee | 305 | 3.1 | Lausanne | 100 | 2.32 | Shepherd and Edmonds [1] |
| Australia | 1978 | 27 | Mallee | 305 | 3.1 | SLS | 60 | 3.15 | Shepherd and Edmonds [1] |
| Australia | 1978 | 27 | Mallee | 305 | 3.1 | Glenfield | 91 | 3.36 | Shepherd and Edmonds [1] |
| Australia | 1978 | 27 | Mallee | 305 | 3.1 | Lausanne | 98 | 3.15 | Shepherd and Edmonds [1] |
| Australia | 1964 | 12 | Gippsland | 594 | 3.3 | SLS | 94 | 1.85 | Shepherd and Edmonds [1] |
| Australia | 1964 | 12 | Gippsland | 594 | 3.3 | Glenfield | 98 | 2.65 | Shepherd and Edmonds [1] |
| Australia | 1964 | 12 | Gippsland | 594 | 3.3 | Lausanne | 100 | 2.11 | Shepherd and Edmonds [1] |
| Australia | 1969 | 18 | Gippsland | 594 | 3.3 | SLS | 90 | 2.12 | Shepherd and Edmonds [1] |
| Australia | 1969 | 18 | Gippsland | 594 | 3.3 | Glenfield | 99 | 2.37 | Shepherd and Edmonds [1] |
| Australia | 1969 | 18 | Gippsland | 594 | 3.3 | Lausanne | 100 | 2.11 | Shepherd and Edmonds [1] |
| Australia | 1974 | 22 | Gippsland | 594 | 3.3 | SLS | 85 | 2.36 | Shepherd and Edmonds [1] |
| Australia | 1974 | 22 | Gippsland | 594 | 3.3 | Glenfield | 98 | 2.65 | Shepherd and Edmonds [1] |
| Australia | 1974 | 22 | Gippsland | 594 | 3.3 | Lausanne | 100 | 2.45 | Shepherd and Edmonds [1] |
| Australia | 1978 | 27 | Gippsland | 594 | 3.3 | SLS | 79 | 2.59 | Shepherd and Edmonds [1] |
| Australia | 1978 | 27 | Gippsland | 594 | 3.3 | Glenfield | 95 | 3.06 | Shepherd and Edmonds [1] |
| Australia | 1978 | 27 | Gippsland | 594 | 3.3 | Lausanne | 98 | 3.15 | Shepherd and Edmonds [1] |
| Australia | 1978 | 27 | Yathong | 384 | 4.2 | Lausanne | 54 | 5.1 | Parer et al. 1994 [9] |
| Australia | 1978 | 27 | Pine Plains | 305 | 3.1 | Lausanne | 71 | 4.65 | Parer et al. 1994 [9] |
| Australia | 1978 | 27 | Urana | 442 | 3.2 | Lausanne | 98 | 3.15 | Parer et al. 1994 [9] |
| Australia | 1983 | 32 | Mallee | 305 | 3.1 | Lausanne | 60 | 4.95 | Parer et al. 1994 [9] |
| Australia | 1984 | 33 | Urana | 442 | 3.2 | Lausanne | 93 | 3.72 | Williams et al. 1990 [8] |
| Australia | 1984 | 33 | Snowy Plains (Sub-alpine) | 1247 | -0.8 | Lausanne | 72 | 4.62 | Williams et al. 1990 [8] |
| Australia | 1984 | 33 | Arid | 225 | 5.4 | Lausanne | 100 | 3.32 | Williams et al. 1990 [8] |
| Australia | 1990 | 39 | Mallee | 305 | 3.1 | Lausanne | 55 | 5.07 | Parer et al. 1994 [9] |
| Australia | 1990 | 39 | Gippsland | 594 | 3.3 | Lausanne | 79 | 4.39 | Parer et al. 1994 [9] |
| Australia | 1990 | 39 | Wimmera | 339 | 4.4 | Lausanne | 55 | 5.07 | Parer et al. 1994 [9] |
| Australia | 1994 | 43 | NSW | 460 | 3.1 | Lausanne | 44 | 5.35 | Fenner and Fantini 1999 [1] |
| Australia | 1995 | 44 | Cooma | 540 | -2 | SLS | 20 | 4.24 | Kerr et al. 2017 [10] |
| UK | 1966 | 13 | Norfolk | NA | NA | Brecon | 90 | 1.03 | Ross and Sanders 1984 [14] |
| UK | 1967 | 14 | Norfolk | NA | NA | Brecon | 94 | 0.76 | Ross and Sanders 1984 [14] |
| UK | 1967 | 14 | Skokholm | NA | NA | Brecon | 97 | 0.43 | Ross and Sanders 1984 [14] |
| UK | 1968 | 15 | Norfolk | NA | NA | Brecon | 86 | 1.23 | Ross and Sanders 1984 [14] |
| UK | 1969 | 16 | Norfolk | NA | NA | Brecon | 84 | 1.32 | Ross and Sanders 1984 [14] |
| UK | 1970 | 17 | Norfolk | NA | NA | Brecon | 59 | 2.08 | Ross and Sanders 1984 [14] |
| UK | 1976 | 23 | Norfolk | NA | NA | Brecon | 21 | 3.12 | Ross and Sanders 1984 [14] |
| UK | 1978 | 25 | Wiltshire | NA | NA | Brecon | 45 | 2.44 | Ross and Sanders 1984 [14] |
| UK | 1979 | 26 | Angus | NA | NA | Brecon | 44 | 2.46 | Ross and Sanders 1984 [14] |
| UK | 1979 | 26 | Wiltshire | NA | NA | Cornwall | 44 | 1.8 | Ross and Sanders 1984 [14] |
| UK | 1980 | 27 | Wiltshire | NA | NA | Wiltshire | 52 | 1.86 | Ross and Sanders 1984 [14] |
| UK | 1978 | 25 | Hants | NA | NA | Brecon | 0 | 4.46 | Ross and Sanders 1985 [14] |

Table S2. Summary of the final linear regression model for the Australian data.

| *Predictor* | *Estimate* | *95% CI* | *p-value* |
| --- | --- | --- | --- |
| (Intercept) | 0.28 | -0.34 – 0.91 | 0.367 |
| Years since SLS release [1st degree] | 5.94 | 3.84 – 8.04 | <0.001 |
| Years since SLS release [2nd degree] | -1.07 | -3.37 – 1.23 | 0.355 |
| Years since SLS release [3rd degree] | 6.26 | 3.87 – 8.64 | <0.001 |
| Years since SLS release [4th degree] | 4.84 | 4.02 – 5.66 | <0.001 |
| Average annual rainfall | -0.86 | -1.42 – -0.30 | 0.004 |

Table S3. Summary of the final linear regression model for the UK data.

| *Predictor* | *Estimate* | *95% CI* | *p-value* |
| --- | --- | --- | --- |
| (Intercept) | -0.860 | -0.906 – -0.814 | 0.041 |
| Years since myxoma virus arrival | 0.138 | 0.038 – 0.237 | 0.011 |
